# Effectiveness of Simple Individual Psychoeducation for Bipolar II Disorder

**DOI:** 10.1155/2016/6062801

**Published:** 2016-07-31

**Authors:** Yuka Saito-Tanji, Emi Tsujimoto, Reiko Taketani, Ami Yamamoto, Hisae Ono

**Affiliations:** ^1^Department of Psychological Science, Graduate School of Humanities, Kwansei Gakuin University, 1-155 Uegahara Ichibancho, Nishinomiya, Hyogo 662-8501, Japan; ^2^Medical Science, Eli Lilly Japan K.K., Sannomiya Plaza Building, 7-1-5, Isogamidori, Chuo-ku, Kobe, Hyogo 651-0086, Japan

## Abstract

Several studies have proven the effectiveness of psychoeducation in bipolar II disorder patients; however, simpler psychoeducation is needed in daily medical practice. Therefore, we devised a simple individual psychoeducation program, which involved 20-minute sessions spent reading a textbook aloud in the waiting time before examination. Here, we report a successful case of simple individual psychoeducation with a patient with bipolar II disorder, a 64-year-old woman who had misconceptions surrounding her mood due to 24 years of treatment for depression. Her perception of mood state, particularly mixed state, was dramatically changed, and her quality of life was improved after the simple individual psychoeducation. This case suggests that the simple individual psychoeducation could be effective for bipolar II disorder by improving understanding of the disease and by meeting different individual needs.

## 1. Introduction

Bipolar II disorder is defined as a clinical course of recurring mood episodes consisting of at least one major depressive episode and at least one hypomanic episode [1]. It has been often considered a mild form of bipolar I disorder, perhaps based on the definition of hypomania, which is a less severe mood elevation compared to mania. However, some studies have found that the course and outcome of bipolar II disorder are worse than those for bipolar I disorder with respect to multiple unfavorable illness characteristics, such as higher rates of relapse [2], more rapid cycling, greater anxiety, and greater risk of suicide attempt [3, 4]. Therefore, bipolar II disorder may have more severe effects on patient quality of life.

Impairment of disease awareness is often found in patients with psychiatric disorder. Patients with bipolar II disorder show especially poor awareness of their illness [5], because a hypomanic episode is often not severe enough to cause serious impairment in social functioning. This low awareness might be a cause of the long time to diagnosis in bipolar II disorder; recent research on a bipolar II cohort showed that the mean time from onset of symptoms to bipolar diagnosis was approximately 20 years [6]. Greater lack of awareness and longer time to diagnosis might constitute psychopathological features that complicate the course of bipolar II disorder.

The most common treatment of bipolar II disorder is medication, including mood stabilizers and antipsychotics [7]. Many clinical studies have been conducted on bipolar I disorder or bipolar disorders that partially included bipolar II disorder, as there is often no clear evidence for bipolar II disorder [7]. However, some studies reported that psychotherapies could help reduce symptoms [8] and prevent relapse [9] in bipolar II disorder.

Psychoeducation has been one of the most investigated psychotherapies [10] and is recommended by several guidelines as a first choice for the maintenance treatment of bipolar disorder [7, 11]. Colom and colleagues reported the efficacy of 21-session group psychoeducation on relapse prevention in patients with bipolar II disorders in a randomized controlled trial [9]. Recently, another randomized study showed that biological rhythm tended to improve in patients with bipolar II disorder after 6 sessions of group psychoeducation [12]. Based on these results, psychoeducation may have advantages in terms of life style regularity and relapse prevention in patients with bipolar II disorder. Psychoeducation could be especially appropriate in patients with bipolar II disorder due to the condition's complicated characteristics, including the greater lack of disease awareness and longer time to diagnosis.

It is difficult to implement a systematic and comprehensive group psychoeducation in out-patient clinics in Japan. One reason may be the lack of economic assistance for psychoeducation healthcare coverage. There may also be insufficient time to perform psychoeducation, because the number of outpatients with mood disorders has been increasing [13]. Despite the positive findings on group psychoeducation in bipolar II disorder [9, 12], some particular issues with group treatment stand out [14]. For example, patients may wait longer for group therapy compared to individual therapy, because it takes time to assemble a group. There is less flexibility in session scheduling, which may cause lower attendance compared to individual therapy.

Therefore, we devised a simple individual psychoeducation program for a patient with bipolar II disorder, a program that is easy to implement in a clinical setting and could be considered worthwhile not only in Japan but also in other countries. The psychoeducational sessions took place in the waiting time before a medical examination. The duration of a session was approximately 20 minutes, basically once a week. In the session, the textbook* Living with Bipolar Disorder* [15], which includes the symptoms, pharmacotherapy, psychotherapy, and possible risk factors of bipolar disorder, was read aloud by the patient together with the a psychotherapist.

In this report, we presented a case of a bipolar II patient with whom the simple individual psychoeducation was very effective. This report was approved by the Kwansei Gakuin University Institutional Review Board for the Protection of Human Subjects of Research, and the work was conducted according to the principles of the Declaration of Helsinki.

## 2. Case Presentation

The patient was a 64-year-old housewife. She had been treated for depression for 24 years. Approximately a year ago, she became so talkative and irritable that she quarreled with medical staff and her family. Because a doctor refused to prescribe the medication she requested, she admitted herself to another hospital. However, she was also uncomfortable with treatment received at this hospital and left on her own recognizance. After discharge, she repeatedly visited the emergency department with complaints of stomach pain. No physical conditions were found, and emergency staff strongly recommended that she see a psychiatric specialist.

She then visited our clinic. At the first medical examination, she was hypomanic, extremely talkative, and somewhat arrogant. She was diagnosed with bipolar II disorder, as assessed by the Diagnostic and Statistical Manual of Mental Disorders (DSM-IV-TR) [1], and this diagnosis was told to her. She responded that she did not care what the disease was called and ordered the doctor to treat her so that she would return to a cheerful and exhilarated state, which was medically diagnosed as hypomanic. Paroxetine was withdrawn, and olanzapine was increased. Even under medication, she visited the doctor almost every day, without an appointment, and repeatedly complained about her medication for a month. After six months, her mood became euthymic, and she was able to keep regular follow-up appointments. However, she demanded to restart paroxetine. She thought that she was still depressed because she was taking the wrong medication. She was therefore recommended for the psychoeducation program.

At the psychoeducation first session, the level of her understanding of bipolar disorder was assessed via a self-made questionnaire. She answered 100% correctly in awareness of bipolar disorder, 66.7% in symptoms, 60.0% in treatment, and 50.0% in pathogenesis. The knowledge regarding bipolar disorder may have been acquired from explanations during medical examinations in the preceding six months. Additionally, her mood was assessed by the Internal State Scale (ISS) [16–18], which is a self-report measure in which 17 items are rated using a visual analog scale from 0 to 100 to discriminate between the mood states based on the scoring algorithm using ISS subscales. ISS indicated her mood as a mixed state (Table 1). However, she insisted that she was always depressed. She claimed that her irritability and quarrels, which were assessed as manic/hypomanic on the ISS, were caused by a depressive state. She said that she felt irritable because she could not do everything as smoothly as before because of depression and that she quarreled with her family because she could not do as well in household affairs because of depression. At this session, she read aloud the section regarding prevalence and diagnosis of bipolar disorder in the textbook. She appeared unwilling throughout the session.

At the second psychoeducation session, she read aloud the section regarding manic/hypomanic states and depression symptoms in bipolar disorder. She could not understand that the hypomanic state was a pathologic state. She still believed that the hypomanic state was an ideal state that she should reach in recovery. She believed that she should talk as much as she did because everyone enjoyed her funny talking. Eventually, she concluded that she did not have bipolar disorder because she had never experienced manic/hypomanic phases as described in the textbook.

At the third psychoeducation session, she read the section regarding mixed states in bipolar disorder and the disease process. Reading the description of mixed states, her perception of her disease was dramatically changed, and she began to think that this description might explain her current mood. She came to understand that her irritability was not due to depression or medication but due to the instability of her mood and the poor communication with her family. She recognized that she annoyed everyone around her when she was talkative and that that was one of the symptoms of the disease. She said, “Psychoeducation has enabled me to confront the disease. I want to know my mood objectively and I want to control it.”

At the fourth psychoeducation session, she read aloud the section describing how to control mood swings in bipolar disorder, the importance of medication, and the treatment goals. She said that she wanted her family to understand the symptoms of bipolar disorder and advise her about her state of mood, because it would be useful to objectively know her mood and control it. During the fifth session, she mainly read the section describing how to control rhythms of daily living, how to deal with daily stress, and the welfare system. She said “I sometimes feel I can work more actively, and I like to go out more often, but I try to keep my daily living rhythm, for example doing housekeeping easily and staying indoors.” She voluntarily started writing a diary to objectively observe her mood. In the sixth to eighth sessions, she read aloud the textbook sections on pharmacological psychological treatments. She seemed convinced of the efficacy of medication with olanzapine, and she stopped asking the doctor to prescribe paroxetine. She continued the diary and controlled daily living as part of the psychotherapy, although she said that she was still depressed.

At the ninth and final session of psychoeducation, she mainly read aloud the sections regarding pathology and recent research on bipolar disorder. She felt relieved to know the biological mechanism of her mood swings. During this session, her understanding of bipolar disorder and her mood were assessed again with the ISS. She answered 33.3% of the questions correctly in awareness of bipolar disorder, 100.0% in symptoms, 100.0% in treatment, and 100.0% in pathogenesis. The ISS indicated that her mood was manic/hypomanic (Table 1), but no discrepancy was found between mood on the ISS and her subjective perception.

## 3. Discussion

We presented a successful case of a simple individual psychoeducation program with a patient with bipolar II disorder, who had misconception about the euthymic state due to long treatment for depression. The psychoeducation program improved her understanding of bipolar disorder and her perception of mood state. As a result, she was more able to communicate appropriately with her family and medical staff, and her quality of life was much improved.

The most marked feature of the case was the 24 years from the first psychiatric visit to a diagnosis of bipolar II disorder. While being treated for depression, the patient was readily given an antidepressant and anxiolytics when she complained that she was not exhilarated. This caused her to think that she was suffering from depression when she did not feel exhilarated. She believed that the hypomanic state with exhilaration was normal and she should be recovered to that state. In addition, her desire to return to the hypomanic state with exhilaration, including a relentless appeal to medical institutions and her family, had caused trouble with interpersonal relationships and reduced her quality of life.

Her general understanding of the disease was not significantly changed after psychoeducation, which was mainly explained because she had already obtained general knowledge of the disease prior to psychoeducation. She had many chances to learn about the disease from her physician during the six months between the first visit to our hospital and the beginning of psychoeducation. However, she actually obtained knowledge of mixed states from the psychoeducation, and she carefully learned the details of mixed states in a short time. One advantage of an individual method is that physicians can formulate a detailed response to patients. There is thus the possibility that individual psychoeducation could be more effective for patients with bipolar II disorder, because patients with bipolar II disorder often have different histories of present illness and different problems.

The ISS indicated that her mood based on ISS indicated was a mixed state at the first session and it was manic/hypomanic at the last session. At the start of psychoeducation, there was a discrepancy between her mood as scored on the ISS and her subjective perception, perhaps because she did not understand mixed states. She was hardly aware of the hypomanic state and strongly recognized the depressive state. In the final psychoeducation session, her mood as scored on the ISS matched her subjective perception, which may have been because she obtained knowledge of the mixed state and opinions about her mood from her family.

The largest success factor in the case was that the patient obtained knowledge of mixed states and became aware that the hypomanic state with exhilaration was not the ideal state. She recognized that the euthymic state, which she had believed to be depression before psychoeducation, was a comfortable state for her social life. This change contributed greatly to her improved quality of life. As many patients with bipolar disorder have gone long periods before receiving a diagnosis of the disorder [19], they may suffer from distorted awareness of the symptoms. In particular, in bipolar II disorder, detection of the disease is often delayed because the hypomanic state is difficult to recognize. Considering this, there are presumably many cases of bipolar disorders, especially bipolar II disorder, in which distortion in symptom recognition occurs, as it did in the present case. Therefore, psychoeducation, which might correct such distortion, should be further considered for the treatment of bipolar disorder in the future.

Individual psychoeducation might also be useful for a wide range of subjects because a patient could proceed at their own speed. The simpler method and the less restricted schedule of individual psychoeducation were also more suitable for patients' attendance. Simple psychoeducation also has an advantage in terms of cost. The medical fee for this psychoeducation was only 3,300 yen (about 27.23 USD) per session. As this psychoeducation requires no special charges, it imposes less of a burden on patients and medical institutions.

There are some limitations in this case report. We developed the questionnaire used to assess the level of understanding of the disorder, and its reliability and validity have not been confirmed. The program's long-term effectiveness was also not assessed. Thus, it is necessary to conduct further trials, including randomized controlled trials.

## 4. Conclusions

We reported that a simple individual psychoeducation program was efficacious in a patient with bipolar II disorder. The recognition of mood in this patient was significantly improved. A simple individual psychoeducation program might be thus useful and highly feasible in a clinical setting to treat bipolar disorder II.

## Figures and Tables

**Table 1:** Internal State Scale (ISS) subscale scores and mood states at first and final psychoeducation sessions.

	First session	Final session
*ISS subscale scores*		
Activation	230	170
Well-being	0	190
Perceived conflict	100	90
Depression index	200	80

*ISS defined mood states*	Mixed	Manic/hypomanic
